# Phylogenomics of species from four genera of New World monkeys by flow sorting and reciprocal chromosome painting

**DOI:** 10.1186/1471-2148-7-S2-S11

**Published:** 2007-08-16

**Authors:** Francesca Dumas, Roscoe Stanyon, Luca Sineo, Gary Stone, Francesca Bigoni

**Affiliations:** 1 Dipartimento di Biologia animale (DBA) Università degli Studi di Palermo, via Archirafi 18. Palermo, Italy; 2 Dipartimento di Biologia Animale e Genetica, Laboratori di Antropologia, Via del Proconsolo 12, 50122 Firenze, Italy; 3 Comparative Molecular Cytogenetics Core, National Cancer Institute, Frederick Maryland, USA

## Abstract

**Background:**

The taxonomic and phylogenetic relationships of New World monkeys (Platyrrhini) are difficult to distinguish on the basis of morphology and because diagnostic fossils are rare. Recently, molecular data have led to a radical revision of the traditional taxonomy and phylogeny of these primates. Here we examine new hypotheses of platyrrhine evolutionary relationships by reciprocal chromosome painting after chromosome flow sorting of species belonging to four genera of platyrrhines included in the Cebidae family: *Callithrix argentata* (silvered-marmoset), *Cebuella pygmaea* (pygmy marmoset), *Callimico goeldii* (Goeldi's marmoset) and *Saimiri sciureus* (squirrel monkey). This is the first report of reciprocal painting in marmosets.

**Results:**

The paints made from chromosome flow sorting of the four platyrrhine monkeys provided from 42 to 45 hybridization signals on human metaphases. The reciprocal painting of monkey probes on human chromosomes revealed that 21 breakpoints are common to all four studied species. There are only three additional breakpoints. A breakpoint on human chromosome 13 was found in *Callithrix argentata*, *Cebuella pygmaea* and *Callimico goeldii*, but not in *Saimiri sciureus*. There are two additional breakpoints on human chromosome 5: one is specific to squirrel monkeys, and the other to Goeldi's marmoset.

**Conclusion:**

The reciprocal painting results support the molecular genomic assemblage of Cebidae. We demonstrated that the five chromosome associations previously hypothesized to phylogenetically link tamarins and marmosets are homologous and represent derived chromosome rearrangements. Four of these derived homologous associations tightly nest *Callimico goeldii* with marmosets. One derived association 2/15 may place squirrel monkeys within the Cebidae assemblage. An apparently common breakpoint on chromosome 5q33 found in both *Saimiri* and *Aotus nancymae* could be evidence of a phylogenetic link between these species. Comparison with previous reports shows that many syntenic associations found in platyrrhines have the same breakpoints and are homologous, derived rearrangements showing that the New World monkeys are a closely related group of species. Our data support the hypothesis that the ancestral karyotype of the Platyrrhini has a diploid number of 2n = 54 and is almost identical to that found today in capuchin monkeys; congruent with a basal position of the Cebidae among platyrrhine families.

## Background

Molecular data have led to a revaluation of the time for primate origins and current views suggest that paleontologists have underestimated the time depth of primate origins [1,2]. Previously most paleontologists thought that the origin of primates was around 60 million years ago (mya) approximately at the Cretaceous/Tertiary (K/T) boundary, but there is a growing consensus that primates probably originated at least 85–90 mya. Fossil evidence indicates an African or possibly an Indo-Madagascan origin for primates, however data for a geographic origin are largely circumstantial [2,3].

It now appears that the division of the two major branches of primates, Strepsirrhini (lemurs and lorisoids) and Haplorrhini (tarsiers, monkeys, apes and humans) would then easily predate 60 mya and probably 77 mya [2,4]. Recent molecular contributions have reinforced the hypothesis of a monophyletic African origin of strepsirrhines [2,4-6]. The division between lorisoids and lemuroids, the two major branches of strepsirrhines, is deep and probably established by 65 mya.

Current paleontological interpretations about anthropoid origins are dependent on where tarsiers are placed in the primate phylogenetic tree. The Strepsirrhini/Haplorhini taxonomic division of primates was based on the morphological conclusions that tarsiers were more closely related to anthropoid primates (monkeys, apes, and humans) than to the "lower" primates [7]. However, the molecular data are ambivalent on tarsier affinities and have been interpreted to link tarsiers with anthropoids [8-10], or to Strepsirrhini [11-14]. Apparently, the strepsirrhine/tarsier/anthropoid divergence was so rapid that for current molecular methods it appears as a trichotomy [2]. There are some isolated fossil teeth from Africa dated between 45 and 60 mya which may be close to the origin of anthropoids, but more complete and diagnostic remains from the Fayum (Egypt) only appear at about 35–37 mya [15] long after the probable origin of anthropoids. The Asian fossils appear to be a sister clade to these African remains and there may be a complicated pattern of multiple primate faunal exchanges between Asia and Africa.

Even if the origin of stem anthropoids has not yet been unequivocally elucidated [16-18] the anthropoid primates surely include both New World (Platyrrhini) and Old World (Catarrhini) monkeys, apes and humans.

### Platyrrhine origins and taxonomy

Various hypotheses have been advanced for the origin of New World monkeys (NWM) and whether they were a mono or a polyphyletic assemblage [19]. The current consensus is that paleowinds, island-hopping and vegetation rafting, favoured a Paleogene African origin of New World monkeys [20,9]. Various dispersal events probably occurred over a 20 million year period making repeated contributions to colonization possible and a plausible case for the multi-phyletic origin of extant NWM [21]. The dentition, particularly M3, of *Branisella boliviana*, at 27 mya the oldest fossil platyrrhine monkey, supports an African origin of NWM because it is similar to fossils from the Oligocene and Late Eocene of Fayum, Egypt [22]. Comparative phylogenomics demonstrates a close phylogenetic relationship between these primates, compatible with a monophyletic African origin for all New World monkeys [23,24].

Dates for the origin of the platyrrhine/catarrhine divergence from the molecular data are considerably earlier than the oldest fossil remains and range from about 35 mya to over 50 mya with 40 mya as a fair compromise [1,25]. A summary of molecular evidence indicates that extant platyrrhines diverged over the last 20 million years [21,26] making it probable that the radiation of living species occurred wholly in the Neotropics to take advantage of favorable ecological opportunities. This radiation produced numerous species with a wide range of morphological, ecological and ethological adaptations. Today these monkeys range from southern Mexico to northern Argentina.

The taxonomic and phylogenetic relationships of these monkeys continue to challenge primatologists and are difficult to define on the basis of morphological characters because of problems in distinguishing homology and convergence [27]. In Platyrrhinae "sibling species" are not uncommon and paleontological data are as yet so limited that they are of little use to resolve phylogenetic relationships. We do not know much about what happened to the platyrrhines between 40 and 20 million years ago and it would be quite helpful to have further paleontological evidence to shed light on this gap [26].

### Traditional taxonomies of Playrrhines and the position of *Callimico goeldii*

**Figure 1:**
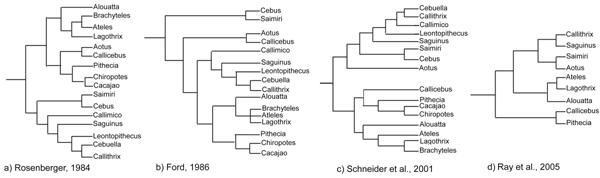
**Four phylogenies for New World monkey genera**. a) modified from Rosenberger et al 1984 [72] and b) modified from Ford 1986 [73] are based on morphological traits, while c) modified from Schneider et al., 2001 [40] and d) modified from Ray et al., 2005 [39] are based on molecular studies. See text for further details.

These difficulties are reflected in the large number of different phylogenetic trees and taxonomies presented by various authors over the years (fig 1). The various treatments of *Callimico goeldii *are explicatory. This species resembles tamarins and marmosets in small body size and claws, but shares with other NWM characters like single births and a third molar. Traditional taxonomies either recognized two or three families of NWMs. When two families were recognized (Cebidae and Callitrichidae) *C. goeldii *was placed into one or the other and was seen as basal to the other species [24,28-30]. Others erected a third family (Callimiconidae) to accommodate *C. goeldii *[31-34]. All families were then placed in one superfamily, Ceboidea. A typical and largely followed morphological based taxonomy is that of Groves (1993)[35] with two families: 1. Callitrichidae, including the genera *Callimico, Callithrix, Leontopithecus, Saguinus*, and 2. Cebidae: including genera *Alouatta, Aotus, Ateles, Brachyteles, Lagothrix, Callicebus, Cebus, Saimiri, Cacajao, Chiropotes, and Pithecia*.

#### Molecular tree of New World monkey phylogeny

Molecular data has led to a radical revision of the traditional divisions and assemblages of NWMs. The separation into two distinct branches of Cebidae and Callitrichidae is not supported. Instead, three clades are distinquished [36-41]:

1. Cebidae, marmosets (including *Callimico*) and tamarins are placed in a clade with *Saimiri*, *Cebus *and probably *Aotus*

2. Pitheciidae which groups *Callicebus, Pithecia*, *Cacajao *and *Chiropotes*.

3. Atelidae which consists of *Alouatta*, *Ateles*, *Lagothrix *and *Brachyteles*.

Indeed, Groves (2001), without providing any evident morphological justification, altered his taxonomy of NWMs probably to reflect developments in primate phylogenomics. His scheme is identical to that above, but erects Nyctipithecidae with the single genus *Aotus *as a fourth family.

On the other hand, the low number of nucleotidic differences found between Platyrrhine species, because the divergence between many taxa was probably short, often limits the resolution of molecular studies. There is some uncertainty as to the exact branching order, both between and within the three taxonomic divisions (fig. 1) [39-42].

### Molecular cytogenetic in Platyrrhine phylogenomics

Comparative molecular cytogenetics represents an independent database to morphology and sequencing for studying New World primate evolution. Chromosome rearrangements are important markers in the evolution of species, because rearrangements are rare genomic events often linked to the speciation process [43]. Over the last decade the use of chromosomes for evolutionary and phylogenetic research has received a particularly strong impulse from molecular cytogenetics. FISH techniques in comparative genomics have proven to be powerful instruments to individuate and study chromosomal changes. Painting probes permitted chromosomal homologies to be established between different species on the basis of DNA content [44] and have uncovered many of the translocations intervening between various species of primates. Initially human probes were produced and hybridized to ape and monkey chromosomes to determine homology at the DNA level. The introduction of Flow activated chromosome sorting (Facs) has widened the production of painting sets from other primates and many species of mammals. The use of "reciprocal painting" has deepened the possibilities of investigation. In fact "reciprocal painting" not only confirms results obtained with FISH in an independent experiment, but also provides new and more detailed information about breakpoints in chromosomal rearrangements. The correct identification of breakpoints determines if similar rearrangements actually involve homologous chromosomal segments. When different breakpoints are detected, it is clear that the rearrangements involved do not derive from the same chromosomal rearrangement. Both cases have different weight in phylogenetic interpretation.

As is commonly seen in arboreal primates, it is well known that the evolutionary rate of chromosomal rearrangements in Platyrrhinae is very high [45-47]. Because rapidly evolving systems usually provide good phylogenetic resolution, our research was focused on using molecular cytogenetic data (chromosome sorting and multi-directional chromosome painting) to help clarify the evolution, taxonomy and phylogenetic relationships between these primates.

Cytogenetics confirms that New World monkey biodiversity is still not well known and that species number is probably underestimated in traditional taxonomy. Molecular cytogenetics data indicate that many taxa including Atelidae (genera, *Lagothrix*, *Brachyteles, Alouatta *and *Ateles*) and other species (*Callicebus, Aotus*) are karyologically derivated [45,47-53].

Chromosome painting has already provided important information about the molecular taxonomy of Platyrrhinae and in particular Cebidae. One hypothesis on the basis of chromosome painting with human paints was that *C. argentata*, *C. pygmaea *and *C. goeldii *(with *Saguinus oedipus *and *Callithrix jacchus*) shared derived chromosomal associations that define the Callitrichidae family [37].

**Figure 2:**
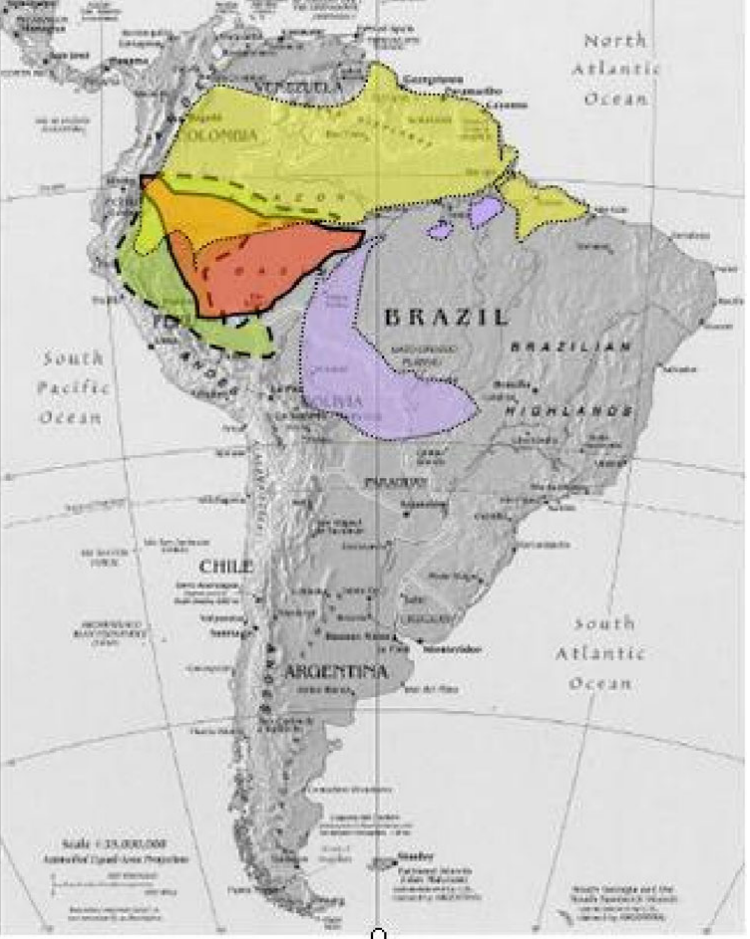
**Geographic distribution of four species of Cebidae**. Geographic distribution of the four species studied by flow sorting and reciprocal chromosome painting: *Callithrix argentata *in violet, *Cebuella pygmaea *in red, *Callimico goeldii *in green and *Saimiri sciureus *in yellow.

**Figure 3:**
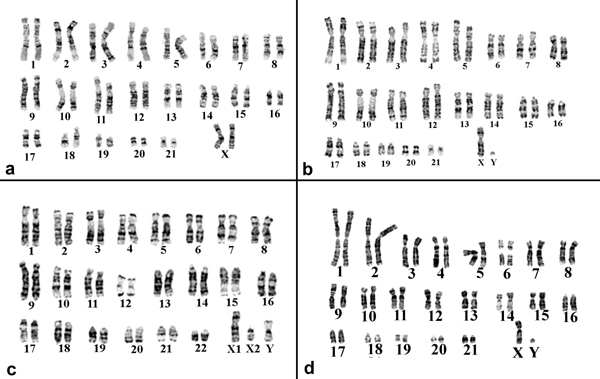
**G-banded karyotype of four species of Cebidae**. a) *Callithrix argentata*, b) *Cebuella pygmaea*, c) *Callimico goeldii *and d) *Saimiri sciureus*. The X-chromosome of *Saimiri sciureus *differs by a pericentric inversion or centromere shift.

We used "reciprocal painting" to study four species of New World monkeys: *Callithrix argentata *(CAR), *Cebuella pygmaea *(CPY), *Callimico goeldii *(CGO) and *Saimiri sciureus *(SSC) (Figure 2). Paints of the chromosomes of these four monkey species (Figure 3) were produced through Facs, and then hybridized to human chromosomes. We integrated our new data with already available information obtained through hybridization of human paints on chromosomes of *C. argentata *(2n = 44), *C. pygmaea *(2n = 44), *C. goeldii *(2n = 46–47) ([37] and on *Saimiri sciureus *(2n = 44) [53].

## Results

### Flow sorting

#### *Callithrix argentata*

**Figure 4:**
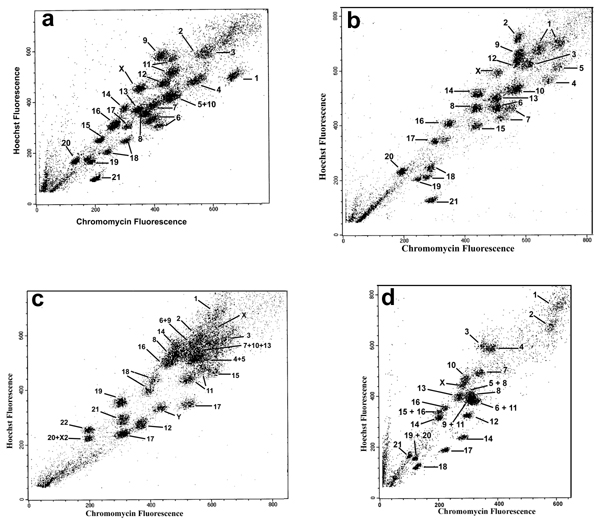
**Flow karyotypes of four species of Cebidae**: a) *Callithrix argentata*, b) *Cebuella pygmaea*, c) *Callimico goeldii *and d) *Saimiri sciureus*.

The bivariate flow karyotype of *Callithrix argentata *was resolved into 25 peaks (Fig. 4a). Flow sorting and degenerate nucleotide primed (DOP)-PCR provided chromosome paints from each peak. These paints were then hybridized to *Callithrix argentata *to identify the chromosome content of each peak. All but one peak provided single chromosomes. Chromosomes CAR5 and CAR10 were sorted together in one peak despite the repetition of sorting experiments. Chromosomes 6, 11 and 18 were each found in two peaks.

#### *Cebuella pygmaea*

The bivariate flow karyotype of *Cebuella pygmaea *was resolved into 24 peaks (Fig. 4b). Flow sorting and DOP-PCR provided chromosome paints from each peak. These paints were then hybridized to *Cebuella pygmaea *to identify the chromosome content of each peak. All peaks provided single chromosomes. Chromosomes 1, 7 and 18 were each found in two peaks.

#### *Callimico goeldii*

The bivariate flow karyotype of *Callimico goeldii *was resolved into 23 peaks (Fig. 4c). Flow sorting and DOP-PCR provided chromosome paints from each peak. These paints were then hybridized to *Callimico goeldii *to identify the chromosome content of each peak. All but 4 peaks provided single chromosomes. Chromosomes 4/5, 6/9, 7/10/13 and 20/X2 were sorted together in one peak despite the repetition of sorting experiments. Chromosome 11 was found in two peaks.

#### *Saimiri sciureus*

The bivariate flow karyotype of *Saimiri sciureus *was resolved into 21 peaks (Fig. 4d). Flow sorting and DOP-PCR provided chromosome paints from each peak. These paints were then hybridized to *Saimiri sciureus *to identify the chromosome content of each peak. All but 5 peaks provided single chromosomes. Chromosomes 5/8, 6/11, 9/11, 15/16 and 19/20 were sorted together in one peak despite the repetition of sorting experiments. Chromosome 16 was found in two peaks.

### Hybridization of monkey paints on human chromosomes

#### *Callithrix argentata* and *Cebuella pygmaea*

**Figure 5:**
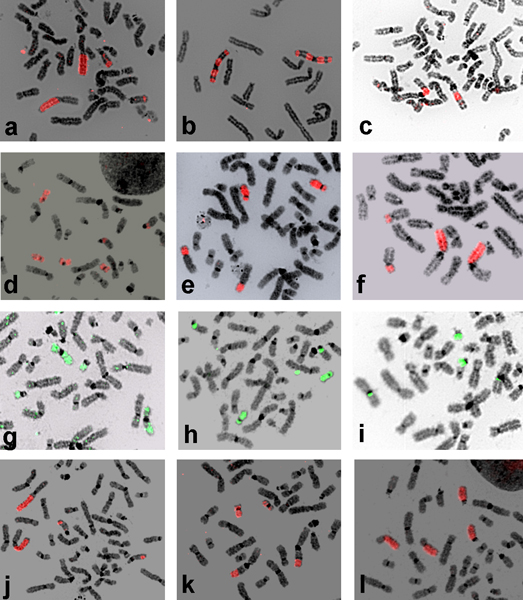
**Hybridization of Cebidae probes on human metaphases**. Hybridization examples of Cebidae probes on a human metaphase: a) CAR 12 paints segments of HSA 2 and 15, b) CAR 15 paints segments of HSA 3, c) CAR 18 paints a segment of HSA 1q, d) CPY 4 paints HSA 17, 20 and a segment of HSA 13, e) CPY 8 paints HSA 8p and 18, f) CPY 10 paints HSA 1p and 10p, g) CGO 15 paints segments of HSA 9 and all of 22, h) CGO 17 paints a segment of HSA 13 and all of 17, i) CGO 22 paints a small segment of HSA 3 and all of 21, j) SSC 7 paints HSA 2q and segments of 15, k) SSC 14 paints a segment of HSA 1q and most of 19, l) SSC 15 paints HSA 8p and 18.

**Figure 6:**
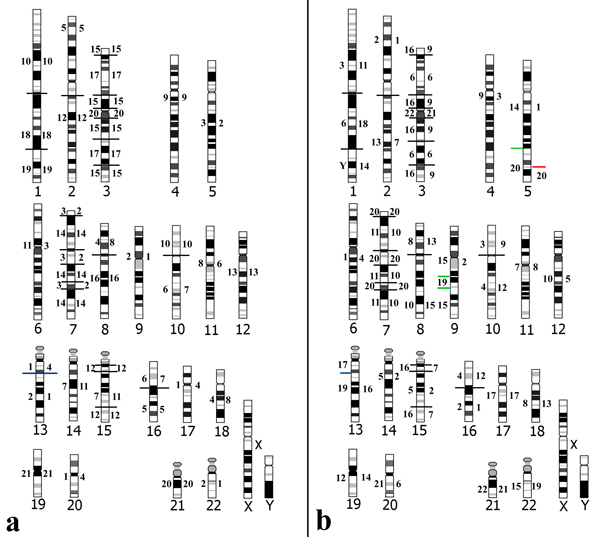
**Human idiogram with hybridization assignments of Cebidae paints**. Human chromosomes are numbered below: a) *Callithrix argentata *chromosomes numbered to the left and to the right, *Cebuella pygmaea *chromosomes; b) *Callimico goeldii *chromosomes numbered to the left and to the right, *Saimiri sciureus *chromosomes.

Both *C. argentata *and *C. pygmaea *paints produced 42 signals on human autosomes (Fig. 5 and Fig 6a). Six marmoset chromosomes have identical syntenies in humans: HSA 4, 6, 11, 12, 19 and the X chromosome. Eight other human chromosomes (5, 9, 14, 17, 18, 20, 21 and 22) were hybridized by a single marmoset chromosome paint. Seven human chromosomes (2, 7, 8, 10, 13, 15 and 16) were hybridized by two marmoset chromosomes. Finally human chromosome 1 and 3 were each hybridized by 3 marmoset probes. The alternating hybridization signals of two or more paints on human chromosomes 3, 7 and 15 suggested that inversions had occurred after divergence of the common ancestor. For chromosomes 3 and 7 it is well known that paracentric and pericentric inversions occurred in the line leading to humans. But it is not known in which line the inversions in chromosome 15 occurred.

#### *Callimico goeldii*

On human autosomes, 45 signals were detected by *C. goeldii *paints (Fig 5 and Fig 6b). Seven *C. goeldii *chromosomes have identical syntenies in humans: HSA 4, 6, 11, 12, 19, 20 and the X chromosome. Five other human chromosomes (14, 17, 18, 21, and 22) were hybridized by a single *C. goeldii *paint, but formed syntenic associations in *C. goeldii*. Nine human chromosomes (2, 5, 7, 8, 9, 10, 13, 15 and 16) were hybridized by two monkey chromosomes. Human chromosomes 1 and 3 were hybridized as above by 3 human paints. Human chromosomes 3, 7 and 9 as above had alternating patterns of two paints indicating inversions which occurred in the human line. Human chromosome 15 also had an alternating hybridization pattern indicating an inversion.

#### *Saimiri sciureus*

On the human autosomes, 42 signals were detected using *Saimiri sciureus *paints (Fig. 5 and 6d). Eight *Saimiri sciureus *chromosomes have identical syntenies in humans: HSA 4, 6, 11, 12, 13, 17, 22 and X. Five other human chromosomes (14, 18, 19, 20, 21) were painted by a single monkey probe, but formed syntenies associations in *Saimiri sciureus*. Two monkey probes painted seven human chromosomes 2, 5, 7, 8, 10, 15 and 16. As previously human chromosomes 1 and 3 were each hybridized by three monkey paints. The probe for SSC 2 painted two entire human chromosomes (HSA 9 and HSA 14) and one segment on HSA 15. Again alternating signals to monkey paints were found on human chromosomes 3, 7 and 15.

### Breakpoints

The reciprocal painting revealed by the hybridization pattern of monkey probes on human chromosomes shows that 21 breakpoints are common to all four studied species. There are only three additional breakpoints. A breakpoint on human chromosome 13 was found in CPY, CAR and CGO, but not in SSC. There are two additional breakpoints on human chromosome 5: one is specific to SSC, and the other to CGO.

Previous research showed that the associations of segments of human chromosomes 9/13, 9/22, 13/17 were present in all studied species of marmosets, tamarins and in *Callimico*. The reciprocal chromosome painting shows that these associations have identical breakpoints and are completely homologous in all these species. The association 2/15 was also reported in these species and in *Saimiri*, but not in other platyrrhines. The reciprocal painting also shows that the segments forming this association are homologous.

## Discussion

The reciprocal painting results, in general, support the molecular genomic assemblage of Cebidae [36-38,40,41]. We demonstrated that the five chromosome associations, which phylogenetically link tamarins and marmosets are homologous and derive from shared chromosome rearrangements in a common ancestor (table 1). Four of these derived homologous associations tightly nest *Callimico goeldii *within the radiation of these species. The reciprocal chromosome painting data confirmed that *Callimico goeldii *is nested within Cebidae as previously hypothesized by Neusser et al 2001 on the basis of unidirectional painting of human chromosome paints. This evidence of homology of derived syntenic associations is important, especially for *C. goeldii*, whose taxonomic position was historically controversial. According to morphological studies, this species was included by some authors in the family Cebidae [7] or placed in Callitrichidae, but in a basal position.

**Table 1:** Number of chromosome segments found in various New World monkey primates.

	APLK	CAP	SSC	CPY	CAR	CJA	CGO	SOE	LSH	ANA
**2n**	54	54	46	44	44	46	46,47	46	46	54
**1**	3	3	3	3	3	3	3	3	3	3
**2**	2	2	2	2	2	2	2	2	2	3
**3**	3	3	3	3	3	3	3	3	3	**4**
**4**	1	1	1	1	1	1	1	1	1	2
**5**	1	1	**2**	1	1	1	**2**	1	1	**3**
**6**	1	1	1	1	1	1	1	1	1	1
**7**	2	2	2	2	2	2	2	2	2	**3**
**8**	2	2	2	2	2	2	2	2	2	2
**9**	1	1	1	1	1	1	**2**	1	1	2
**10**	2	2	2	2	2	2	2	2	2	2
**11**	1	1	1	1	1	1	1	**1**	1	2
**12**	1	1	1	1	1	1	1	1	1	1
**13**	1	1	1	**2**	**2**	**2**	**2**	1	1	1
**14**	1	1	1	1	1	1	1	1	1	**2**
**15**	2	2	2	2	2	2	2	2	2	**6**
**16**	2	2	2	2	2	2	2	2	2	2
**17**	1	1	1	1	1	1	1	1	1	**2**
**18**	1	1	1	1	1	1	1	1	1	1
**19**	1	1	1	1	1	1	1	1	1	1
**20**	1	1	1	1	1	1	1	1	1	1
**21**	1	1	1	1	1	1	1	1	1	1
**22**	1	1	1	1	1	1	1	1	1	1
**X**	1	1	1	1	1	1	1	1	1	1
**Y**	1	1	1	1	1	1	1	1	1	1
**Seg**	34	36	41	35	35	35	37	35	35	47
**S. A**.	6	6	15	12	12	11	14	11	11	19

The top row lists the species belonging to the family Cebidae: APLK = ancestral platyrrhine karyotype, CAP = *Cebus apella*, SSC = *Saimiri sciureus*, CPY = *Cebuella pygmaea*, CAR = *Callithrix argentata*, CJA = *Callithrix jacchus*, CGO = *Callimico goeldii*, SOE = *Saguinus oedipus*, LCH = *Leontopithecus chrysomelas*, ANA = *Aotus nancymae*. In the left column, 2n is the diploid number of the species. The numbers refer to human chromosomes and under each species the number of segment found in that species, not including segments fragmented due to inversions. Numbers in red show derived fissions. Seg refers to the total number of segments including those produced by inversions. S. A. refers to the total number of syntenic associations found in each species including multiple associations due to inversions. [37, 53, 74–76].

One derived association 2/15 was also found in *Saimiri *linking these species with the Cebidae assemblages. However, the molecular data indicate a closer relationship between *Saimiri *and *Cebus*, another genus of this assemblage. There is no reciprocal painting data on species of the genus *Cebus*. However, the human chromosome painting pattern on *Cebus *species studied up to now suggest that this species maintains a karyotypes almost identical to the ancestral platyrhinne karyotype (APLK). Therefore there are no derived association syntenies cytogenetic data to link *Cebus *with marmosets, tamarins or *Saimiri*. If the molecular data has correctly placed *Saimiri*, then this species shares a common ancestor with *Cebus *after the divergence of the marmosets and tamarins, and the 2/15 association may represent a convergence. Further testing with higher resolution molecular cytogenetics methods (i.e. BAC clones) and sequencing of breakpoints will be needed to determine whether the 2/15 association found in *Saimiri *is truly homologous with that found in marmosets.

**Figure 7:**
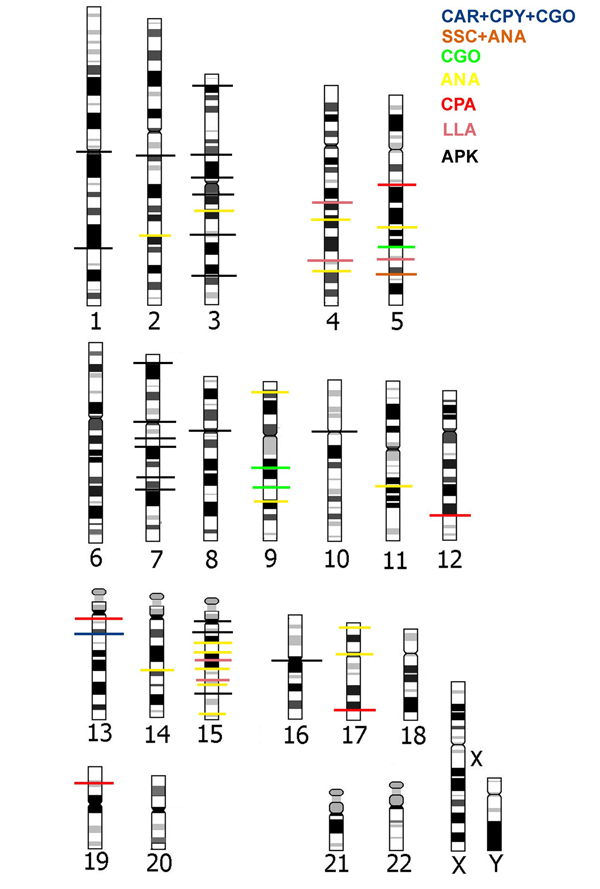
**Human idiogram with all reciprocal chromosome painting data**. Human idiogram with all reciprocal chromosome painting data integrating results from this report and the literature [50, 52, 54] Breakpoints common to all platyrrhine species and hypothesized to be present in the APLK (ancestral platyrrhine karyotypes) are shown as a black line across human chromosome. Breakpoints found in other groups of species or single species are color coded. CPY = *Cebuella pygmaea*, CAR = *Callithrix argentata*, CJA = *Callithrix jacchus*, CGO = *Callimico goeldii*, SSC = *Saimiri sciureus*, ANA = *Aotus nancymae*, CPA = *Callicebus pallescens*, LLA = *Lagothrix lagotricha*.

An apparently common breakpoint on chromosome 5q33 found in both *Saimiri *and *Aotus nancymae *could be evidence of a phylogenetic link between these species (fig 7). In *Saimiri *it forms a separate syntenic association with 5/7 to form SSC 20. In *Aotus nancymae *this segment, along with regions homologous to 4 other human chromosomes (10/11/7/5), forms ANA 4. By integrating our current results with previous reports on reciprocal painting between humans and Platyrrhines, we can show that other fissions of human syntenies 4, 5, 9, 15 and 17 involve different breakpoints and are not homologous.

A 1/3 syntenic association found in both *Aotus nancymae *and *Callimico goeldii *at first appears to be a possible phylogenetic link between these species (table 2). However, both these species have high rates of chromosome evolution making convergence an alternate hypothesis. Our reciprocal painting of *Callimico *paints on human metaphases compared to previous data from reciprocal painting between humans and *Aotus *[54] allows us to confirm the hypothesis that this association is not homologous and results from convergence. The association in *Callimico *can be defined as t1b:3b(1q21-1q32:3p24-3p14, 3q22-3q26.3) while that of *Aotus *is defined as t1c:3c/21(1q32-qter:3p12-q13.1/21).

**Table 2:** Syntenic chromosome associations of segment homologous to human chromosomes found in at least two species of Cebidae.

	2/16	3/21	5/7	8/18	10/16	14/15	1/10	9/13	9/22	13/17	2/15	17/20	1/3	n. apo
**APLK**	X	X	X	X	X	X								
**CPY**	X	X	X	X	X	X	X	X	X	X	X	X		
**CAR**	X	X	X	X	X	X	X	X	X	X	X	X		
**CJA**		X	X	X	X	X	X	X	X	X	X	X		
**CGO**	X	X	X	X	X	X	X	X (2x)	X	X			X	2
**SOE**	X	X	X	X	X	X		X	X	X	X	X		
**LCH**	X	X	X	X	X	X		X	X	X	X	X		
**SSC**	X	X	X	X	X	X					X			5
**CAP**	X	X	X	X	X	X								
**ANA**		X	X	X	(X)	X							X	14

The top row lists the syntenic associations of segment homologous to human chromosomes found in at least two species of Cebidae. The last column (n. apo), reports the number of additional apomophic associations. APLK = ancestral platyrrhine karyotypes, CAP = *Cebus apella*, SSC = *Saimiri sciureus*, CPY = *Cebuella pygmaea*, CAR = *Callithrix argentata*, CJA = *Callithrix jacchus*, CGO = *Callimico goeldii*, SOE = *Saguinus oedipus*, LCH = *Leontopithecus chrysomelas*, ANA = *Aotus nancymae *[37, 53, 74–76].

There is a previous report that provides some weak support for a cytogenetic link between *Callicebus *and *Aotus *provided by the syntenic association 10/11 [50]. However, the reciprocal painting in these species cannot determine if the rearrangements are homologous. The orientation and exact fusion points between HSA 10 and 11 in both taxa need to be tested with BAC FISH and sequencing to determine if this syntenic association is equivalent or different.

It can also be noted that the assignment of *Aotus *with the Cebidae is not as solid as for other species. A sister relation between *Aotus *and the *Cebus/Saimiri *clade is favored by parsimony analysis, but not by other analyses [41]. *Saimiri *was linked to *Aotus *by only 1 Alu insertion and then this branch to *Callithrix *and *Saguinus *by only 2 insertions [39]. Recently, a Bayesian analysis of 59.8 kbp genomic data for 13 primates concluded that the deepest node within the Cebidae was between squirrel monkeys and marmosets at 17.1 mya with 20.8 mya for crown platyrrhine node. Although Singer et al. also claim that Alu elements were able to resolve Cebidae branching, all branches were supported by a maximum of one marker; hardly reassuring [55].

### The ancestral karyotype and monophyly of platyrrhines

Previous reconstructions of the ancestral primate karyotype (APK) hypothesized a diploid number from 2n = 48 to 50 [56-59]. Recent reciprocal chromosome painting has also refined the content of the APK. Both Stanyon et al. (2006)[60] and Nie et al. [61](2006) found in loriforms a syntenic association 7/16 identical to that found in the proposed ancestral eutherian karyotype [62]. Therefore 7b/16p should be included in the APK. Defined by reference to homology with the human karyotype, the genome of the last common ancestor of all living primates had the following chromosomes:

1, 2a, 2b, 3/21, 4, 5, 6, 7a, 7b/16b, 8, 9, 10, 11, 12a/22a, 12b/22b, 13, 14/15, 16a, 17, 18, 19a, 19b, 20, X and Y.

Then from the APK the origin of the anthropoids was marked by: 1) a fission of the syntenic association 7b/16b, 2) a reciprocal translocation that gave origin to 12 and 22, 3) fusion of 19p and 19q. The ancestral anthropoid karyotype common to both Old World and New World primates maintained a diploid number of 2n = 46 but with the following chromosomes: 1, 2a, 2b, 3/21, 4, 5, 6, 7a, 7b, 8, 9, 10, 11, 12, 13, 14/15, 16a, 16b, 17, 18, 19, 20, 22, X e Y.

The New World monkeys all share and are characterized by seven fissions in five chromosomes (1, 3/21, 8, 10 and 14/15) and by 4 fusions, which form syntenic associations (2b/16a, 5/7a, 8b/18 and 10b/16b). A comparison of our reciprocal painting with that available in the literature shows that all these syntenic associations have the same breakpoints and are homologous derived rearrangements (Figure 7). Additional, indirect, supporting evidence for the homology of these breakpoints across platyrrhine species comes from the multidirectional painting of *Sanguinus oedipus *and *Lagothrix lagothricha *paints on various species [63] including Atelidae [64,65]. In all these cases the painting revealed numerous conserved autosomal syntenies compatible with an origin in a common ancestor. Therefore, our data and that of the literature support the hypothesis that the ancestral karyotype, (as reported in Stanyon et al 2003, 2004) of the New World monkeys has a diploid number of 2n = 54: 1a, 1b, 1c, 2a, 2b/16a, 3a, 3b, 3c/21, 4, 5/7a, 6, 7b, 8a, 8b/18, 9, 10a 10b/16b, 11, 12, 13, 14/15a, 15b, 17, 19, 20, 22, X and Y.

### Divergence order of Platyrrhines

Molecular cytogenetic data do not provide convincing evidence on the order of divergence between platyrrhine families. However, it is suggestive that the karyoytpe found in the genus *Cebus *is almost identical to that of the APLK for diploid number (2n = 54) and for associations. It differs only for a pericentric inversion and heterochromatin additions. This condition is congruent with a basal position of the Cebidae.

Both Canavez et al. (1999)[36], Schneider et al. (2001)[40] and Seuanez et al (2005)[66] also placed the Cebidae as basal with the Pitheciidae and Atelidae sharing a more recent common ancestor. Analysis of primate retroviral restrictive domains also placed the Cebidae as basal [67]. Ray et al. (2005)[39] on the basis of *Alu *insertions places the Pitheciidae as basal and with the Cebidae and Atelidae sharing a more recent common ancestor. Steiper and Ruvolo (2003)[41] using G6PD data depending on the type of analysis put either Atelidae or Pitheciidae as basal. In a recent study of orthologous sequences of six nuclear genes compared for most platyrrhine genera, the branching order between the three clades shifted when different algorithms were used. In one Pitheciidae were basal with Cebidae and Atelidae sharing a more recent common ancestor; in the other the Cebidae were basal with Atelidae and Pitheciidae as sister clades [42]. For now, it seems that molecular studies apparently cannot yet unequivocally determine the relationship between Cebidae, Atelidae and Pitheciidae.

## Conclusion

Our results support the molecular genomic assemblage of Cebidae. Five chromosome associations, phylogenetically linking tamarins and marmosets are homologous and derive from shared chromosome rearrangements in a common ancestor. Four derived homologous associations nest *Callimico goeldii *within the radiation of the marmosets. One derived association 2/15 may link *Saimiri *with these species. An apparently common breakpoint on chromosome 5q33 may link *Saimiri *and *Aotus*. A comparison of our reciprocal painting with that available in the literature shows that the great majority of syntenic associations and breakpoints found in New World monkeys are homologous. Our data support the hypothesis that the ancestral karyotype of all Neotropical primates had a diploid number of 2n = 54 with chromosomes: 1a, 1b, 1c, 2a, 2b/16b, 3a, 3b, 3c/21, 4, 5/7a, 6, 7b, 8a, 8b/18, 9, 10a 10b/16b, 11, 12, 13, 14/15a, 15b, 17, 19, 20, 22, X and Y. This suite of derived chromosome rearrangements found in all these monkeys, overwhelming supports the monophyly of NWM and shows that these primates form a tight phylogenetic and taxonomic unit. Although molecular cytogenetic data do not yet provide convincing evidence on the order of divergence between platyrrhine families, it does suggest that the conserved karyotypes found in species of the genus *Cebus *is congruent with a basal position of the Cebidae.

However, it should be noted that chromosome painting even with reciprocal hybridizations does not usually detail intrachromosomal rearrangements and breakpoint resolution is limited. Cloned DNA probes such as BACs, cosmids and locus specific probes, provided increased resolution and can reveal intrachromosomal rearrangments and precisely map breakpoints. Further high resolution molecular cytogenetic research, using such cloned DNA probes, will be necessary to confirm and resolve unanswered questions of New World primate evolution [68-70]. These questions are urgent today because many of these primates are highly endangered.

## Methods

### Cell samples, tissue culture and chromosome preparation

Metaphase preparations were obtained from established fibroblast cell lines of one male individual of *Callimico goeldii *and of *Cebuella pygmaea*, of one female individual of *Callithrix argentata *and of one male individual of *Saimiri sciureus*. The cell lines were kindly provided by S. O'Brien, Laboratory of Genomic Diversity, National Cancer Institute, Frederick MD, USA. Normal culture procedures were followed. Cultures were maintained in DMEM supplemented with 10% fetal bovine serum.

### Flow sorting and *in-situ *hybridization

Chromosome-specific probes from the NWM were made by DOP-PCR from flow sorted chromosomes using PCR primers amplification and labeling conditions as previously described [44,71]. Chromosome sorting was performed using a dual laser cell sorter (FACSDiVa). This system allowed a bivariate analysis of the chromosomes by size and base-pair composition. About five hundred chromosomes were sorted from each peak in the flow karyotype. Chromosomes were sorted directly into PCR tubes containing 30 μl of distilled water. The same 6MW primer was used in the primary reaction and to label the chromosomal DNA with biotin dUTP or digoxigenin-dUTP (both from Roche) in a secondary PCR for indirect detection. Direct labeling was with rodamine 110-dUTP (Perkin-Elmer) for green, Spectrum Orange (Vysis) for red and Cy5-dUTP (Amersham) for infrared as previously described [57]. *In situ* hybridization and probe detection were carried out following common FISH procedures. About 300–400 ng of each PCR product per probe, together with 10 μg of human Cot-1 (Invitrogen) were precipitated and then dissolved in 14 μl hybridization buffer. After hybridization and washing of the slides, biotinylated DNA probes were detected with avidin coupled with fluorescein isothiocyanate (FITC, Vector, Burlingame, CA). Digoxigenin-labeled probes were detected with antidigoxigenin antibodies conjugated with Rodamine (Roche, Eugene, Oregon).

Digital images were taken using a cooled Photometrics CCD camera coupled to the microscope. Imaging software was SmartCapture (Digital Scientific, Cambridge, UK).

## Competing interests

The authors declare that they have no competing interests.

## Authors' contributions

RS designed the experiments. FD, GS and RS carried out the experiments. All authors participated in analysing the data. FB, FD, RS and LS wrote the paper.
